# A correction method for systematic error in ^1^H-NMR time-course data validated through stochastic cell culture simulation

**DOI:** 10.1186/s12918-015-0197-4

**Published:** 2015-09-04

**Authors:** Stanislav Sokolenko, Marc G. Aucoin

**Affiliations:** Department of Chemical Engineering, University of Waterloo, 200 University Avenue West, Waterloo, N2L 3G1 ON Canada

**Keywords:** NMR, Metabolomics, Time-course, Cell culture, Quantification, Systematic error, Stochastic simulation, Internal standard, Dilution

## Abstract

**Background:**

The growing ubiquity of metabolomic techniques has facilitated high frequency time-course data collection for an increasing number of applications. While the concentration trends of individual metabolites can be modeled with common curve fitting techniques, a more accurate representation of the data needs to consider effects that act on more than one metabolite in a given sample. To this end, we present a simple algorithm that uses nonparametric smoothing carried out on all observed metabolites at once to identify and correct systematic error from dilution effects. In addition, we develop a simulation of metabolite concentration time-course trends to supplement available data and explore algorithm performance. Although we focus on nuclear magnetic resonance (NMR) analysis in the context of cell culture, a number of possible extensions are discussed.

**Results:**

Realistic metabolic data was successfully simulated using a 4-step process. Starting with a set of metabolite concentration time-courses from a metabolomic experiment, each time-course was classified as either increasing, decreasing, concave, or approximately constant. Trend shapes were simulated from generic functions corresponding to each classification. The resulting shapes were then scaled to simulated compound concentrations. Finally, the scaled trends were perturbed using a combination of random and systematic errors. To detect systematic errors, a nonparametric fit was applied to each trend and percent deviations calculated at every timepoint. Systematic errors could be identified at time-points where the median percent deviation exceeded a threshold value, determined by the choice of smoothing model and the number of observed trends. Regardless of model, increasing the number of observations over a time-course resulted in more accurate error estimates, although the improvement was not particularly large between 10 and 20 samples per trend. The presented algorithm was able to identify systematic errors as small as 2.5 % under a wide range of conditions.

**Conclusion:**

Both the simulation framework and error correction method represent examples of time-course analysis that can be applied to further developments in ^1^H-NMR methodology and the more general application of quantitative metabolomics.

**Electronic supplementary material:**

The online version of this article (doi:10.1186/s12918-015-0197-4) contains supplementary material, which is available to authorized users.

## Background

Hydrogen nuclear magnetic resonance (^1^H-NMR) spectroscopy is an emerging tool for metabolomic analysis of cell culture. In contrast to the established use of ^13^C-NMR for targeted elucidation of intracellular metabolic flux (reviewed in [1]), the quantification of a broader cellular metabolome with ^1^H-NMR in the context of recombinant protein production has been much more recent [2–6]. Unlike ^13^C-NMR, which requires relatively expensive ^13^C labelled compounds and often complex interpretation, ^1^H-NMR benefits from simple sample preparation and non-selective data acquisition. The result is that a single scan can reveal the concentration of many small molecules in an unbiased manner, with concentration levels reaching as low as the micromolar range. Despite the maturity of ^1^H-NMR technology, the context of cell culture metabolomics offers opportunities for further developments in both acquisition and post-processing of metabolomic time-course data.

Quantitative NMR relies on the principle that the integrals of resonance peaks are proportional to the number of nuclei that make up the resonances [7]. The absolute area of the integrals is also dependent on spectrometer and sample properties that include the relaxation time of various metabolites, pulse excitation, and broad-band decoupling. While the effect of relaxation time can be ignored with a sufficiently long acquisition time (or measured and factored in directly – see [7]), the effect of other factors is accounted by comparison to a calibration standard. Typical calibration standards can be broadly categorized as internal (where a known quantity of a compound is added directly to the sample), external (where a known quantity of a compound is scanned in a co-axial tube), or electronic (where a synthetic signal generated inside the NMR is used as reference) (see [8] for an in-depth review). Regardless of how the reference signal is generated, metabolite quantification relies on the ratio of target resonance and reference peak integrals. Unlike typical measurement variability, error in the generation or measurement of the reference signal will have the same relative impact on all the quantified metabolites and represents one example of a systematic bias.

Error related to the reference standard can stem from sample preparation (in the form of pipetting) as well as spectra processing and analysis. Although external and electronic standards do not rely on the addition of a chemical standard, the lack of internal standard introduces extra variability from the amount of sample analyzed. Proper technique can ensure good reproducibility, but occasional mistakes are nonetheless possible. More importantly, the reference peak is subject to the same variability as any other resonance. Phase and baseline correction, which are typically performed on all NMR spectra, are known to have a considerable impact on the accuracy of peak area integration [9]. Malz and Jancke [10] have observed that while routine standard deviation can be reduced to 1.5 % of mean concentration, the relative uncertainty can be as high as 11 % with just “slightly” wrong phase and baseline corrections. Other factors may also come into play depending on the quantification method. Some commercial packages such as Chenomx NMR Suite (Chenomx Inc., Edmonton, Canada), which has been used in recent cell culture applications [3–6], require the user to match the observed internal standard peak to an idealized representation. Apart from introducing user uncertainty, this method may be particularly sensitive to line shape variability. Discrepancies between the ideal and observed shapes of the internal resonance peak due to imperfect shimming are a likely source of quantification error.

While errors from standard quantification impact practically all NMR samples to some extent, biofluid and cell culture samples are also subject to dilution effects. Urine samples vary in their water content, which is corrected by normalization to either total spectrum area or a reference metabolite such as creatinine (reviewed in [11] and [8]). The metabolomic analysis of cell lysates, common to many cell culture applications such as drug discovery [12], suffers from similar problems due to the variability of extraction efficiency. The effect of variable solvent concentration results in the same systematic error as from reference quantification – a global underestimation or overestimation in the relative concentrations of all observed metabolites in a given sample.

The application of NMR spectroscopy and other metabolomic approaches to time-course samples presents both a unique challenge and opportunity in dealing with systematic errors. On the one hand, a single biased sample can skew the trends of multiple compounds and suggest false metabolic relationships. On the other hand, the time-course trends of metabolite concentrations have a significant degree of implicit replication that can be exploited through mathematical means. Recent work with cell culture [13] and biofluid [14] data has used nonparametric curve fitting techniques to model metabolite concentration trends by leveraging the inherent smoothness of biological trends. This work extends the concept by identifying systematic deviations across a number of metabolites. In the same way that a dramatic deviation from an overall trend of a metabolite’s concentration is identified as measurement error via smoothing spline regression, the deviations of many metabolites in one sample can be identified as the result of reference error or a dilution effect.

**Fig. 1:**
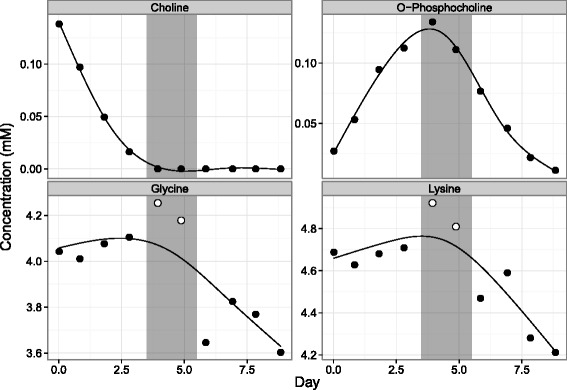
An example of 4 metabolite trends from a metabolic study. Jumps in glycine and lysine concentration trends (highlighted as white points) were hypothesized to be the result of choline exhaustion (region highlighted in grey). Time-course data was collected as described in the Cell culture subsection of the Methods section

In the context of cell culture process monitoring, a subset of compound concentration trends from a batch culture shown in Fig. 1 illustrates the confusion that can arise from possible systematic errors (details provided in the Cell culture section of Methods). The jumps in concentrations of glycine and lysine on days 4 and 5 correspond with the exhaustion of choline and the peak of o-phosphocholine concentration. The question is whether these deviations from the general trend of the compounds can be interpreted as a physiological shift in cellular metabolism or if they are more likely to be the result of systematic error that is associated with internal standard addition. This work presents a simple iterative smoothing algorithm as a means to address this issue. The method is tested by the stochastic generation of cell culture trends subject to simulated observation error to ensure that identified systematic errors are independent of measurement uncertainty.

## Methods

### Cell culture

Metabolic data presented in this work originated from an insect cell media supplementation experiment. *Spodoptera frugiperda* (Sf9) cells were grown in shake flasks at 27 °C and 130 RPM using in-house supplemented IPL-41 media [15]. The cells were routinely split to 0.5 ·10^6^ cells/mL upon reaching a concentration of 2 ·10^6^ cells/mL, with experiments carried out on cells that have undergone less than 30 passages. A 1 L mother flask was seeded at 0.5 ·10^6^ cells/mL with a working volume of 250 mL and grown up to 2 ·10^6^ cells/mL. This flask was used to seed 125 mL flasks at 0.5 ·10^6^ cells/mL with a working volume of 30 mL. Cells were counted and sampled for NMR every 24 h until reaching their maximum concentration (of approximately 7 ·10^6^ cells/mL). 1 mL samples of cell culture media were collected and centrifuged for 8 min at 250 g, with the supernatant collected and stored at - 80 °C until NMR analysis.

The experimental data used as a template for stochastic trend generation, hereafter referred to as reference data, consisted of 4 different carbohydrate supplemented flasks cultured over a period of 10 days. The cultures were identical and seeded from the same stock, but with varying concentrations of glucose and maltose. 43 compounds were profiled for a total of 172 model trends across the 4 flasks. Although many of the compound concentration trends were similar across the flasks, the use of different conditions resulted in more general trends than would be available from replicates.

### NMR

The collected supernatant samples were thawed at room temperature and NMR samples prepared by the addition of 70 uL internal standard to 630 uL supernatant. The standard consisted of 5 mM 4,4-dimethyl-4-silapentane-1-sulfonic acid (DSS) and 0.2 % w/v sodium azide preservative dissolved in 99.9 % *D*_2_O (Chenomx Inc., Edmonton, Canada). The NMR sample solutions were vortexed and pipetted into 5 mm NMR tubes (NE-UL5-7, New Era Enterprises Inc., Vineland, NJ). Samples were randomized and scanned over a two day period on a Bruker Avance 600 MHz spectrometer with a triple resonance probe (TXI 600). Scans were performed using the first increment of a 1D-NOESY pulse sequence with a 1 s presaturation pulse, 100 ms mixing time, and a 4 s acquisition. The acquired spectra were re-randomized [16] and analyzed using Chenomx NMR Suite 7.7 (Chenomx Inc., Edmonton, Canada). Phasing and baseline correction were done automatically by the software and adjusted by a human profiler. Compound concentrations were calculated using the “targeted profiling” method (see [17] for more information). Briefly, the observed spectra were fit by the overlay of idealized NMR resonance peaks from the software library, with compound concentration quantified by comparison to an idealized fit of the DSS resonance peak.

### Systematic error correction

**Fig. 2:**
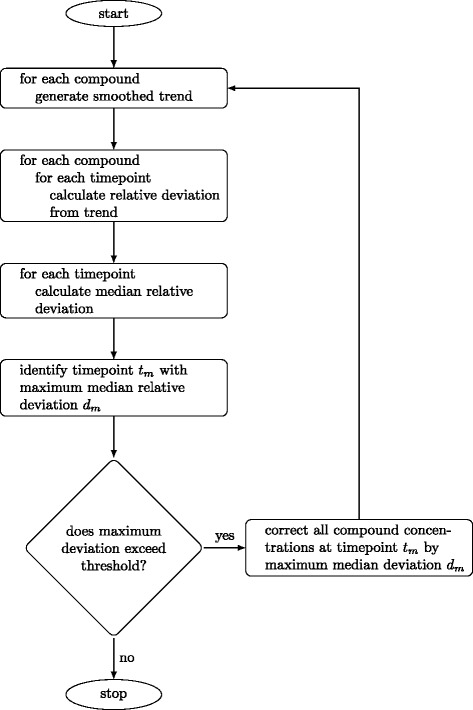
Algorithm flowchart. Step by step description of the internal standard error correction algorithm. Corrected values can be kept or flagged for further investigation/removal

Starting with all compound concentration time-courses from a single cell culture, a nonparametric (smoothing) model was fit to each time-course. Percent deviations from the fits were calculated at each timepoint and for each compound, *ε*_*time*=*i*,*compound*=*j*_=(*y*_*i*,*j*,*observed*_−*y*_*i*,*j*,*smoothed*_)/*y*_*i*,*j*,*smoothed*_. A median percent deviation was taken at each timepoint, corresponding to sorting all the deviations at a given timepoint from lowest (*ε*_*time*=*i*,1_) to highest (*ε*_*time*=*i*,*n*_), and focusing on the middle (or median) value (*ε*_*time*=*i*,*n*/2_). If the largest median percent deviation exceeded a specified threshold, it was subtracted from the observed concentrations of all compounds at the corresponding timepoint. The process was repeated until the largest deviation failed to exceed the specified threshold. An overview of the algorithm is presented as a flowchart in Fig. 2. An R function implementation of the internal standard error correction algorithm is available in Additional file 1.

In principle, the algorithm takes advantage of the fact that an error in internal standard addition or quantification will result in a deviation for all quantified compounds relative to their concentration. As the percentage error from measurement uncertainty can be quite high for some media components [18], the median of relative deviations was chosen as a conservative statistic that could still be capable of identifying systematic error. Mean values were also tested but found to be more susceptible to random noise. An iterative process was used to account for the effect an erroneous measurement can have on a smoothing trend. Once a systematic deviation is identified, the deviating timepoint is corrected and the trend re-smoothed to calculate new deviations. Although the elimination of a deviating timepoint would also be suitable, correction has been chosen in this work as it conserves more of the observed data in the form of a consensus between all compound trends.

The choice of smoothing model and median deviation threshold are two important parameters for error detection. A smoothing model should be chosen according to the expected smoothness of compound concentration trends i.e. how likely they are to exhibit rapid fluctuations. A high-density cell culture or one subject to perturbation may require less smoothing to ensure that rapid physiological changes are not mistaken for internal standard error. On the other hand, a slow-growing or continuous culture could use a much greater degree of smoothing. The median deviation threshold represents the minimum amount of deviation that can be attributed to come from systematic error rather than random measurement uncertainty. High measurement uncertainty is reflected in the variability of median deviation, requiring higher thresholds to prevent false bias detection. However, a number of other factors can also have an impact, including the number of observed compounds and the number of timepoints included in the trend. The effect of these factors on the threshold is explored in this work using stochastic trend generation.

### Stochastic trend generation

The development of a framework for stochastic generation of extracellular compound concentration trends was based on the need to estimate the variability of median relative deviations from a smoothing fit. Trend simulation was reduced to four general parameters – overall trend shape, maximum compound concentration, percent change in compound concentration, and measurement variability. The framework was developed around a reference of collected data and consisted of four steps. First, the reference trends were classified as either increasing, decreasing, concave, or approximately constant. A parametric model was chosen for each classification, and representative curves generated with a domain and range of 0 to 1. The combination of simulated maximum compound concentrations and percent changes were used to generate maximum and minimum concentration values to scale the trend. Finally, measurement variability was simulated and applied to the data. The combination of multiple trends with varying parameters was taken to be a representative of the data one would collect from the time-course of a single culture and is termed “an experiment” throughout the text. R functions used to implement this process are available in Additional file 2 (with an example experiment simulation at the end of the file).

#### Trend classification

Initial classification of the reference data identified trends with a net change in concentration greater than 10 %. Concentrations with changes of less than 10 % were taken as having approximately constant concentrations, or “unclassified”. Simple linear regression was used to classify trends as either increasing or decreasing if the slope was found to be statistically different from 0 at a 95 % confidence level using a t-test. Compound concentrations that had a statistically significant increase followed by a statistically significant decrease were classified as concave (none of the trends could be statistically determined as convex). Trends were left unclassified if the classification of a compound differed across the different experimental conditions. This was done to ensure that classification was restricted to general patterns rather than singular observations. In this way, 15 compounds were classified as decreasing, 14 increasing, 2 concave, and 12 were left unclassified. To allow changes in the number of simulated compounds, these numbers were reformulated and rounded to 35 %, 30 %, 5 % and 30 % of the total compounds respectively.

#### Trend shape

**Fig. 3:**
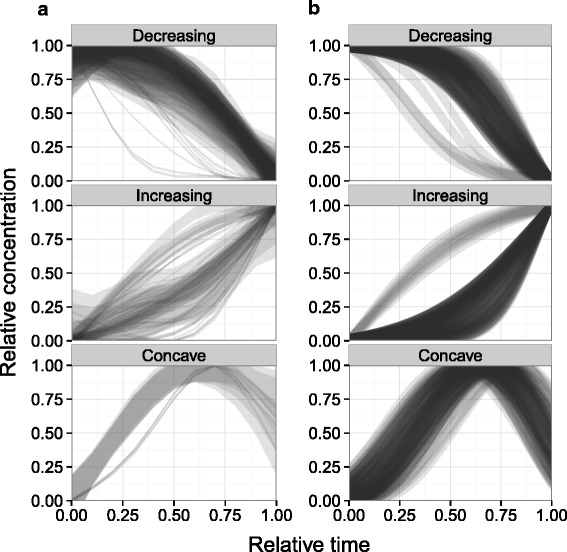
Trend shape simulation. A comparison of **a** observed and **b** simulated compound concentration trends mapped to a domain and range of 0 to 1. Observed data has been smoothed using cubic regression splines with a maxium of 4 degrees of freedom. The line widths represent estimated standard error ranges from the regression spline model. Simulated trend generation is described in the text and the line widths have been set to a constant 5 % of maximum value. Lines were plotted using a high degree of transparency, with darker areas indicating a higher density of curves passing through them. In **b**, 100 trends were generated for each of the classifications using Eqs. 1–2 and parameter values listed in Table 1

Classified reference data trends were smoothed using cubic regression splines with an upper limit of 4 degrees of freedom (Fig. 3a). When normalized to the same domain and range, most of the concentration trends appeared to take very similar shapes. Sigmoidal equations (with 2 parameters) were used to model the increasing/decreasing trends while the concave curves were approximated by a truncated beta distribution density function:

(1) $$ {\fontsize{8}{12}{\begin{aligned}  \textrm{sigmoidal decrease:}\,\,\,\, & y = \frac{1}{1 + e^{\frac{x-a}{b}}}\qquad\quad x \in\, [0, 1] \end{aligned}}}  $$

(2) $$ {\fontsize{8}{12}{\begin{aligned} \textrm{sigmoidal increase:}\,\, \,\,& y = 1 - \frac{1}{1 + e^{\frac{x-a}{b}}} \qquad x \in\, [0, 1]  \end{aligned}}}  $$

(3) $$ {\fontsize{8}{12}{\begin{aligned} \,\,\,\,\,\,\quad\textrm{concave:}\,\,\,\, & y = x^{a-1} \cdot (1-x)^{b-1} \quad x \in\, [c \ge 0, d \le 1] \end{aligned}}}  $$

The sigmoid functions were defined over a domain of 0 to 1, while the beta function’s domain was kept variable. The extra parameters offered greater flexibility in controlling the rate of concentration changes. The y values (and beta distribution x values) were scaled to a range of 0 to 1 after simulation for easier comparison. Unclassified compounds were assumed to follow a linear trend with equal probability of either increasing or decreasing. The linear trend was used to convey a lack of information rather than a strictly linear relationship in compound concentration i.e. the case where a true trend was dwarfed by relative measurement error.

Model parameter ranges were selected by trial and error to visually match the observed trends. As the increasing/decreasing trends showed evidence of two distinct patterns each, two sets of parameters were chosen for the sigmoidal curves along with a separate parameter that related the probability of sampling from one population or the other. The parameters in Table 1 were used to generate the trends in Fig. 3b. Overall, the simulated trends were highly comparable to the observed ones. Although there was less agreement between the concave trends, parameter constraints were kept flexible to account for the low number of concave reference curves.

**Table 1:** Parameter ranges used in the trend shape simulation of reference data (via Eqs. 1-3). Parameter values were drawn from a uniform distribution constrained to the given ranges. Where two ranges are given, the range was chosen randomly for each trend based on the given probability

Trend	Parameter	Range 1	Range 2	P(Range 1)
Sigmoidal decrease	*a*	0.200–0.600	0.600–0.900	0.05
	*b*	0.100–0.180	NA	1.00
Sigmoidal increase	*a*	0.045–0.055	0.945–0.955	0.15
	*b*	0.200–0.400	0.100–0.300	0.15
Concave	*a*	3.500–4.500	NA	1.00
	*b*	2.500–3.500	NA	1.00
	*c*	0.000–0.200	NA	1.00
	*d*	0.800–0.900	NA	1.00

#### Trend range

**Fig. 4:**
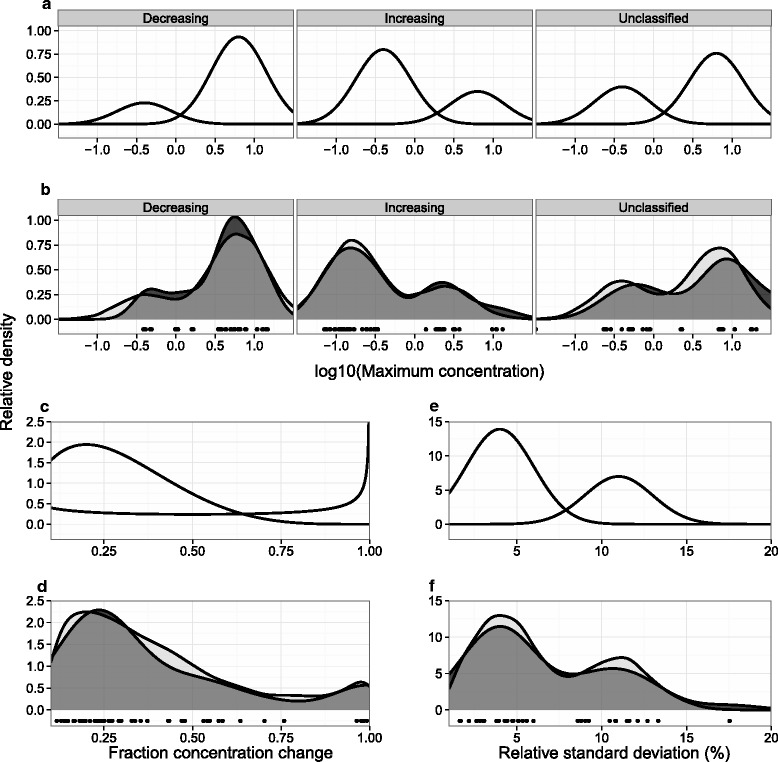
Trend scale and perturbation simulation. **a** Probability density functions used to simulate maximum compound concentration distributions. **b** A comparison of observed (darker grey) and simulated (lighter grey) maximum compound concentration distributions (with a semi-transparent overlap). **c** Probability density functions used to simulate fraction concentration change distribution. **d** A comparison of observed (darker grey) and simulated (lighter grey) fraction concentration change distributions (with a semi-transparent overlap). **e** Probability density functions used to simulate fraction relative standard deviation distribution. **f** A comparison of observed (darker grey) and simulated (lighter grey) relative standard deviation. The curves represent kernel density estimates, with the simulated data generated from 10000 samples per classification from mixtures of two normal distributions (see text for details). Observed data points are shown below the curves

The conversion of idealized trend shapes to realistic concentration time-courses required the generation of minimum and maximum values. The distribution of maximum compound concentrations from the reference data is shown in Fig. 4b. Compounds increasing in concentration were observed to have lower maximum concentrations than decreasing ones, requiring the simulation to be based on trend classification (with concave compounds being treated as increasing). On a logarithmic scale, the spread of maximum concentrations was reasonably modelled by a mixture of two normal distributions with means of -0.4 and 0.8 (corresponding to approximately 0.4 mM and 6.3 mM respectively) and standard deviations of 0.35. The probability density functions of the resulting distributions can be seen in Fig. 4a with the comparison to observed values in Fig. 4b. The proportions between the lower and higher concentration clusters were chosen as 0.20, 0.70, and 0.35 for the decreasing, increasing, and unclassified trends respectively. Although a greater degree of fine tuning was possible to achieve better agreement between observed and simulated distributions, the marginal improvement did not warrant deviating from more general consistency.

To avoid dealing with the correlation between maximum and minimum concentrations (for compounds with relatively small changes in concentration), minimum values were generated from the simulation of net concentration change as a fraction of maximum value. Relative concentration changes were assumed to be less dependent than minimum concentrations on maximum values. As compounds with increasing concentrations were generally observed to have an initial concentration of approximately 0, their percent change was taken as 100 % for the purpose of the simulation. The distribution of fractional changes for decreasing compound concentrations is shown in Fig. 4d. One compound was practically exhausted in all 4 of the tested conditions, with the remainder of the compounds being consumed to various degrees but clustering around 25 % reduction. No change of less than 10 % can be observed as this value had been chosen as a cutoff for separating compounds with a significant trend. The simulation distribution was modelled by a mixture of two beta distributions – one to represent the distribution of non-exhausted compounds (*α*=2, *β*=5) and another to increase the probability of values close to 0 and 1 (*α*=0.5, *β*=0.5), with the proportion between the two set to 0.7 (Fig. 4c). The simulated distribution was truncated to the range of 0.1–1.0 to reflect the reference data. Figure 4d suggests that the simulation was in good agreement with the reference data.

#### Measurement variability

A measurement variability distribution was developed from our previous work on estimating ^1^H-NMR measurement uncertainty for cell culture applications [18]. Briefly, a Plackett-Burman design was used to generate a series of media-like formulations with an orthogonal combination of high and low compound concentrations. In this way, measurement standard deviations for each compound could be estimated independently of other compound concentrations. The result was a collection of relative standard deviations (otherwise referred to as the coefficients of variation) for all compounds in the media. Relative standard deviations for compounds with a statistically significant change in concentration during cell growth were estimated at both high and low concentrations; a single estimate was used for compounds without a significant change.

As the differences in relative standard deviation between compound concentrations were not typically large, all of the relative standard deviations were pooled together into a single distribution of measurement uncertainty (Fig. 4f). Three of the compounds that were particularly challenging to quantify in [18] (and had correspondingly high uncertainties) were excluded as they were not representative of typical quantification – compounds identified to have low concentrations and considerable resonance overlap were not quantified in this work. The resulting distribution took the shape of a bimodal normal distribution (Fig. 4f) with means of 4 % and 11 % and a common standard deviation of 2 % (probability density function shown in Fig. 4e).

### Algorithm validation

The simulation framework was applied to answer two fundamental questions. What is the minimum level of bias that can be identified given normal measurement variability? How is bias identification impacted by the choice of smoothing model and experimental parameters? Two smoothing models were considered – local linear least squares regression and a cubic regression spline. The former was implemented by the loess function in base R and the latter as a general additive model (gam) provided by the mgcv package [19]. Both models made use of a smoothing parameter. The loess approach required a span that dictated what fraction of data points to use in local regression. This parameter was varied from 2.0 (less smooth) to 0.5 (more smooth). The gam approach required the choice of basis dimension number, which was varied from 3 (less smooth) to 6 (more smooth). In the text, models are referred to by their smoothing parameter i.e. loess-0.5 or gam-6. Combined with model type and smoothing parameter, the number of quantified compounds (20–60) and the number of observed data points (10–20) were also seen as important factors that could influence bias detection.

1000 experiments were simulated for each factor combination (with the number of trends making up a single experiment varied as a parameter). Half of the experiments were subject to normal measurement variability, while half were further perturbed with a systematic bias of 5 % at a single randomly selected timepoint. Algorithm performance was assessed by smoothing the simulated data using a given model and calculating the median relative deviation of observations from the fit for each timepoint in each experiment. The result was a pool of median values for each timepoint corresponding to a certain factor combination. Full R code is available in Additional files 3 and 4 for loess and gam simulations respectively.

### Implementation

The algorithms and all analysis has been implemented in the R programming language [20]. Figures were generated using the ggplot2 package [21].

## Results and discussion

### Application

**Fig. 5:**
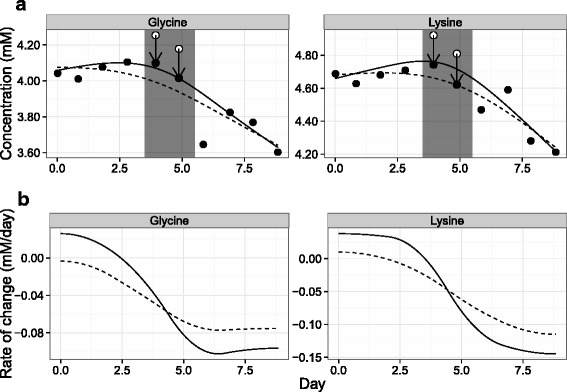
Correction applied to example data. **a** White points represent initially observed concentrations that were marked for correction by the algorithm, while black points represent final compound concentrations (with arrows signifying correction). Smoothing lines were generated using the gam-5 model using uncorrected (solid line) and corrected (dashed line) data. Time-course data was collected as described in the Cell culture subsection of the Methods section. **b** Derivatives calculated from the uncorrected (solid line) and corrected (dashed line) smoothed fits

The correction algorithm was applied to the example data from Fig. 1 and the results can be seen in Fig. 5. Although only glycine and lysine results are shown, all 43 observed compounds were used in the calculation (using a gam-5 smoothing model and a threshold of 2.5 % median deviation). The algorithm provided strong evidence that the jumps in glycine and lysine concentration were not due to metabolic shifts but were the result of a systematic error. Figure 5a also demonstrates that random measurement error such as the pronounced deviation in glycine concentration on day 6 was not impacted by the correction as it was not general to all metabolites. The influence of the correction was most pronounced in the rates of concentration changes calculated as the derivatives of the smoothing curves (Fig. 5b). As a result of the changes in concentration, both compounds went from being produced then consumed to a steady pattern of increasing consumption. More importantly, the correction of only two points resulted in considerable changes to derivative estimates across all time-points. This can have an important impact on the use of spline smoothing for flux estimation in metabolic flux analysis (as in [13], for example).

### Validation

#### Smoothing bias

**Fig. 6:**
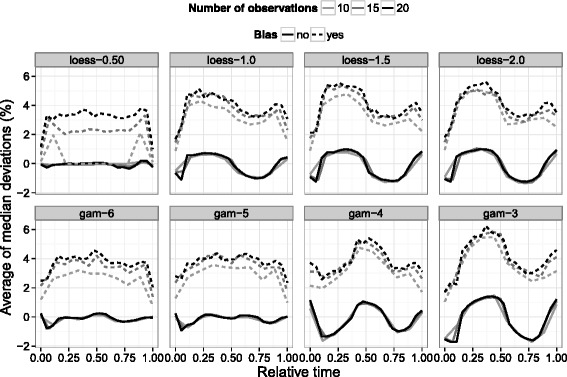
Average bias as a function of smoothing model. Lines represent averages of simulated median relative deviations from smoothing fits. Dashed lines are used to distinguish timepoints simulated with a 5 % bias. The gam-6/loess-0.5 models correspond to a greater degree of smoothing in comparison to gam-3/loess-2.0. The number of observations corresponds to the number of timepoints in the generated metabolic trends. Although the number of compounds per time-course set was varied in the simulation, these were found to have no impact on average trends

The smoothing model used in the correction algorithm must strike a balance in having enough flexibility to follow metabolism related changes in compound concentrations while avoiding undue influence from deviating observations. A lack of flexibility can result in systematic deviations from a smoothing fit where no errors are present, while too much flexibility can underestimate deviations due to error. The simulated trends described in the Algorithm validation section were smoothed using loess and gam models (with varying smoothness parameters) and the median deviations from each experiment were averaged to identify overall trends (Fig. 6). Unsurprisingly, a greater degree of smoothing resulted in less biased deviations i.e. loess-0.5 and gam-6/gam-5 models had practically constant deviations across all timepoints. On the other hand, using an inadequate amount of smoothing generally resulted in an underestimated fit early in the culture (positive deviations from the smoothing fit) and an overestimated fit later. Between the two smoothing functions, gam was found to have a better discrimination of artificially biased timepoints than loess at comparable smoothing levels (gam-5/6 and loess-0.5) – the deviations were more consistent across different timepoints and were not as sensitive to the number of observations. Although the jump from loess-0.5 to loess-1.0 in Fig. 6 is quite considerable, further analysis using other span parameters reinforced the observation that gam smoothing is superior for bias discrimination. As gam-5 requires less information than gam-6, it can be seen as a good compromise between an unbiased fit and deviation identification. For best results, the smoothing model should be tailored to the data under study.

Apart from smoothing model, the number of observations over the course of a culture was also found to have an influence on deviation estimation (Fig. 6). Increased sample frequency yielded a more accurate deviation estimate for biased timepoints. However, the net impact of having a greater number of observations remained quite small. For gam-5, for example, a true bias of 5 % was estimated as approximately 4 % with 15 or 20 observations and closer to 3.5 % with only 10 observations. Further simulations on lower observation numbers suggested that comparable performance could be attained down to 8 observations before degrading to a significant degree (data not shown). As batch processes may be operational for as few as 5 days, this translates to a required sampling frequency of two samples a day. Since 12 h sampling may not always be practical, the effect of a staggered sampling on the correction algorithm was also investigated. With gam-5 smoothing, little to no difference was observed between even 12 h sampling and a routine where 2 samples are taken 8 h apart, followed by a break of 16 h (data not shown).

#### Confidence intervals

**Fig. 7:**
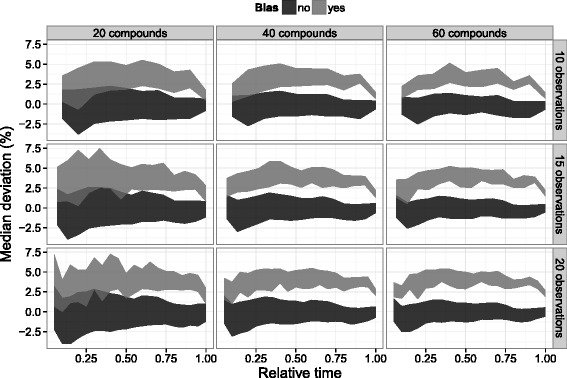
90 % confidence interval around median deviations. Empirical 90 % confidence intervals were constructed from the simulated data by excluding the 5 % highest and 5 % lowest median deviations at each timepoint. Lighter grey colour is used to distinguish timepoints simulated with a 5 % bias

The variability of median deviations is particularly important for the selection of a correction threshold. The threshold must be high enough to avoid correcting deviations due to random measurement noise while remaining sensitive to systematic sources of error. Empirical 90 % confidence intervals were constructed from the simulated data by excluding the 5 % highest and 5 % lowest median deviations at each timepoint (Fig. 7). Between the number of compounds and the number of observations, only the number of compounds was found to have an effect on confidence interval width. Naturally, the observation of more compounds reduced the impact of measurement noise and allowed for a more robust median estimate. However, the simulation of more compounds assumed equal quantification quality. If the number of observed compounds is increased by profiling highly convoluted or otherwise poorly quantifiable compound resonances, the beneficial impact is likely to be limited.

Based on the results, the observation of 40 compounds at 10 timepoints (typical of the data obtained in our lab) will exhibit a natural variation in median deviation of approximately 2–2.5 %. Thus, deviations beyond this threshold have a high probability of occurring due to a source of bias such as internal standard addition or quantification. The results also show that a 5 % bias is more likely to be identified as anywhere between 2.5–5 % (with further reduction in performance at earlier timepoints), meaning that a subtraction of the estimated median deviation is more likely to dampen the bias, rather than remove it. Reduced performance at the end-points reflects the relative lack of trend data and can be ameliorated by adding replicates or extending the observation time beyond the span of direct interest.

#### Simulation extension

To determine how robust the correction method is to changes in the underlying data, four modifications to the simulated data were considered. The ratio of decreasing to increasing trends (intially taken as 35 %:30 % based on our cell culture data) was set to 60 %:5 % as well as 5 %:60 %. Despite these dramatic shifts, both average bias and confidence interval trends remained very similar to those presented in Figs. 6 and 7. The only exception was at the end points, where lower concentration magnitudes resulted in much more variable relative deviations. Since increasing and decreasing trends reach their minimum concentrations at different endpoints, the overall effect on median relative deviations is not pronounced when the two trends are balanced in number. However, the extreme case of a 12:1 imbalance between increasing and decreasing trends resulted in larger variability ranges at time-course edges. With 60 % of the trends increasing, the bias threshold at early timepoints increased from 2.5 % to 5 %. With 60 % of the trends decreasing, the bias threshold increased at late timepoints but did not go beyond the overall average of 2.5 % (as the threshold at these timepoints was already low). The difference between the two conditions can be explained by the fact that all increasing trends start at or very close to 0, while only some of the decreasing trends reach such low concentrations. Since a more balanced proportion of increasing and decreasing trends is expected in real data, the overall effect would be minimal. Two other conditions – increasing the net concentration changes of decreasing trends (by doubling the proportion of compounds with large relative changes) and increasing the variability of observations (by doubling the standard deviation of highly variable compounds) did not appear to have any impact on the threshold calculation. For all conditions, gam-5 smoothing remained the best choice.

Taken together, these results suggest that a bias threshold of approximately 2.5 % using gam-5 smoothing would be an adequate default choice for diverse data sets. Beyond cell culture applications, we predict the bias correction algorithm to be just as useful for other time-course metabolomic data. One such example is biofluid analysis in toxicology. The Consortium for Metabonomic Toxicology (COMET) has already established a large collection of time-course urine samples that meet the requirements for systematic error correction [22]. While the proposed correction is not designed to replace standard normalization techniques, it can build on the development of recent smoothing spline techniques [14] and serve a complementary role in the identification of spurious results. Further extension to mass spectrometry (MS) methods is also likely to be fruitful. Techniques such as multiple reaction monitoring (MRM) are commonly used for pharmaceutical and toxicological metabolomics [23, 24] and suffer from similar dilution effects as NMR (exacerbated by the need for more sample manipulation such as liquid extraction steps). The correction of systematic biases may serve to reduce the relative standard deviations of quantified compound concentrations.

## Conclusions

The growing popularity of quantitative metabolomics for time-course applications presents a new context for data processing and acquisition. While this work deals primarily with the correction of internal standard quantification in cell culture data, it’s not difficult to imagine similar approaches applied to other analytical methods. Improvements in accuracy, precision, and analysis speed can be best achieved by leveraging the replication inherent to the parallel observation of multiple metabolite trends. The algorithm presented in this work took advantage of inherent autocorrelation to identify and correct systematic bias originating from internal standard addition and quantification. The gam-5 model was identified as the best smoothing function for the task, with the ability to detect a bias greater than 2.5 % across most of a culture’s time-course. The simulation framework followed the context-driven approach by capturing the key elements of a cell culture time-course. Although the presented validation has focused on trends typically observed in our lab, full code has been provided to allow rapid adaptation to user needs.

## Availability and requirements

- **Project name:** metcourse
- **Project home page:**https://github.com/ssokolen/metcourse
- **Operating system:** Platform independent (tested on Linux)
- **Programming language:** R (version 3.2.1)
- **Other requirements:** R packages – dplyr (version 0.4.2), mgcv (version 1.8–6)
- **License:** Apache (version 2.0)
- **Any restrictions to use by non-academics:** no

## Additional files

Additional file 1
**Internal standard error correction algorithm.** R script containing two function definitions – f_smooth() used to generate the smoothing fit and correct_relative_deviation() to perform the corrections. (R 2.56 KB)

Additional file 2
**Stochastic trend generation functions.** R script containing all the functions used to simulate realistic timecourse trends. There is an example at the end of the file that shows how the functions are combined to generate a trend. (R 17.6 KB)

Additional file 3
**Code used to generate data for loess analysis.** R script used in this work for generating loess model data. (R 10.5 KB)

Additional file 4
**Code used to generate data for gam analysis.** R script used in this work for generating gam model data. (R 10.5 KB)
